# A level III sentinel lymph node in breast cancer

**DOI:** 10.1186/1477-7819-4-31

**Published:** 2006-06-07

**Authors:** Kim Bowers, Yiyan Liu, Nasrin Ghesani, Steve H Kim

**Affiliations:** 1 Department of Surgery, New Jersey Medical School, University of Medicine and Dentistry, Newark, New Jersey, USA; 2 Department of Radiology, New Jersey Medical School, University of Medicine and Dentistry, Newark, New Jersey, USA; 3 Department of Surgery, Medical Science Building, G-512, 185 South Orange Ave, Newark, NJ 07103, USA

## Abstract

**Background:**

For accurate nodal staging, all blue and radioactive lymph nodes should be sampled during the sentinel lymph node biopsy for breast cancer. We report a case of anomalous drainage in which one of the sentinel lymph nodes was unexpectedly found in the level III axillary space.

**Case presentation:**

A 40-year-old female underwent mastectomy for extensive high-grade ductal carcinoma in-situ (DCIS) with micro-invasion. The index lesion was located in the right upper inner quadrant. Lymphoscintigraphy was performed on the morning of surgery. Two sentinel lymph nodes were identified. At operation, 5 mls of isosulfan blue dye was injected at the same site of the radio-colloid injection. The first sentinel lymph node was found at level I and was blue and radioactive. The second sentinel node was detected in an unexpected anomalous location at level III, medial to the *pectoralis minor*. Both sentinel nodes were negative.

**Conclusion:**

Sentinel node staging can lead to unexpected patterns of lymphatic drainage. For accurate staging, it is important to survey all potential sites of nodal metastasis either with preoperative lymphoscintigraphy and/or rigorous examination of regional nodal basins with the intra-operative gamma probe.

## Background

Sentinel lymph node biopsy has become the standard of care in staging the clinically negative axilla in patients with breast cancer. Although its role in patients with ductal carcinoma-in-situ is controversial, most would agree that it is indicated in the patient being described, as she had both micro-invasion as well as multifocal disease requiring mastectomy (the latter indication being justified in case unexpected invasive cancer is discovered at a site different than that of the index lesion)[1,2]. For accurate nodal staging, removal of all sentinel nodes is important not only to guide postoperative therapy but also for regional control of disease should these nodes contain metastases. Prior to sentinel node biopsy, the standard of care for nodal staging has been level I and II axillary node dissection. The advent of this new technique has now afforded the ability to ultra-stage a limited number of nodes with intense multi-section analysis as well as immunohistochemistry [3]. Furthermore, detection of nodes with radioactivity is now able to identify draining nodes in unexpected locations that would otherwise have been missed by standard axillary node dissection.

## Case presentation

**Figure 1:**
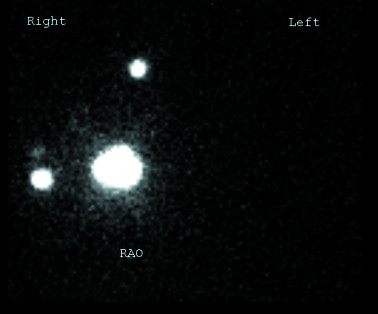
Lymphoscintigraphy demonstrating the location of the primary tumor in the upper inner quadrant of the right breast (large central area of radioactivity) as well as the two sentinel lymph nodes.

**Figure 2:**
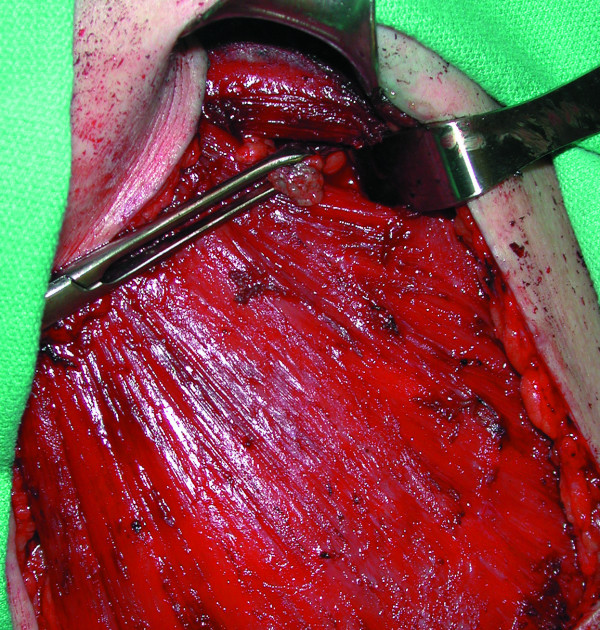
A blue-stained and radioactive level III sentinel lymph node is demonstrated at the end of the Allis clamp. This was dissected via a transpectoral approach.

A 40-year-old female presented with suspicious increasing micro-calcifications in the right breast. A stereotactic biopsy of the most worrisome area in the right upper inner quadrant demonstrated ductal carcinoma *in-situ *with microinvasion. Because of the diffuse extent of the malignant appearing calcifications, a mastectomy was recommended. On the morning of surgery, a 0.4 mCi dose of filtered Tc-99 m sulfur colloid was injected subdermally at the biopsy site. Two sentinel lymph nodes were identified, one at level I and a second, that was very medial and interpreted as a possible internal mammary node (Figure 1). In the operating room, 5 ml of isosulfan blue dye was injected subdermally near the biopsy site, and the procedure was started after 5 minutes of skin massage. The first sentinel lymph node was found at level I and was blue and radioactive. Continued dissection at level I did not demonstrate any other radioactive or blue-stained lymph nodes. However, using the lymphoscintigraphy as our guide, we performed a more thorough search for the second sentinel node in the internal mammary chain and under the most medial part of the clavicle. Radioactivity was detected in the latter location, suggesting a level III sentinel lymph node, i.e., medial to the *pectoralis minor*. Since it was not easily accessible through the small axillary incision, we proceeded with the mastectomy then dissected the remaining sentinel node through a trans-pectoral approach. The medial aspect of the *pectoralis major *was divided in the direction of its fibers, thus allowing access to the level III space medial to the *pectoralis minor*, where a blue and radioactive lymph node was identified and removed (Figure 2). The patient had an unremarkable postoperative course and was discharged on the first post-operative day. Final pathology demonstrated multifocal micro-invasive comedo-DCIS in the upper inner, upper outer, and retro-areolar regions of the breast. Both sentinel lymph nodes were negative for metastatic disease based on routine histology as well as immunohistochemistry for cytokeratin.

## Discussion

Studies of lymphatic drainage patterns from the breast have demonstrated that up to 19% of cases will demonstrate sentinel node activity outside the expected level I and II basins of the axilla (i.e., lateral to the medial border of the *Pectoralis minor*) [4]. Although the incidence of *isolated *metastatic disease to level III (medial to the *Pectoralis minor*) is < 2%, the likelihood of tumor involvement at this level is significantly increased if there are concomitant positive nodes in levels I and II [5,6]. Therefore, it is mandatory that all identified sentinel nodes be removed for complete staging as well as regional control of disease. In this case, sentinel node detection allowed more accurate complete staging since the level III node would not have been included in a standard level I and II axillary node dissection. Because the patient was undergoing mastectomy, access to the level III node was available through the transpectoral approach described. Bevilacqua *et al*., have proposed a management algorithm for patients in whom the approach to the anomalous sentinel node may be technically more difficult than in our patient, i.e., those undergoing local excision rather than mastectomy, or those with an internal mammary sentinel node [7]. The first situation is that of simultaneous sentinel nodes in the expected axillary location as well as in an anomalous location. In this instance, the former are dissected and sent for frozen section analysis. If the axillary sentinel nodes are positive for metastatic disease, then a standard axillary dissection is performed, and the anomalous node is not removed if it is in the internal mammary location (or can be included in the axillary lymphadenectomy if it is at level III). If, however, the axillary sentinel node is negative for metastatic disease or if no axillary sentinel node is identified, then efforts must be made to retrieve the anomalous sentinel node so that the patient may be accurately staged.

## Conclusion

Our case illustrates the degree to which sentinel lymph node biopsy has improved the ability to stage the patient with breast cancer. Not only can intense   histological and immunohistochemical analysis be limited to a small   number of nodes that have the highest probability of containing  metastases, but it also allows identification of first echelon draining   nodes that would normally be missed with a standard axillary node   dissection.

## Competing interests

The author(s) declare that they have no competing interests.

## Authors' contributions

**SK **designed the study, **KB **and **SK **drafted the manuscript. **KB, YL, NG**, and **SK **all contributed to critical revisions and intellectual content.

All authors read and approved the final manuscript.
